# Biochemical and molecular features of chinese patients with glutaric acidemia type 1 from Fujian Province, southeastern China

**DOI:** 10.1186/s13023-023-02833-z

**Published:** 2023-07-26

**Authors:** Jinfu Zhou, Guilin Li, Lin Deng, Peiran Zhao, Yinglin Zeng, Xiaolong Qiu, Jinying Luo, Liangpu Xu

**Affiliations:** 1 Medical Genetic Diagnosis and Therapy Center, Fujian Key Laboratory for Prenatal Diagnosis and Birth Defect, Fujian Maternity and Child Hospital College of Clinical Medicine for Obstetrics & Gynecology and Pediatrics, Fujian Medical University, Fuzhou, 350001 Fujian Province China; 2 Department of Preventive Medicine, School of Public Health, Fujian Medical University, Fuzhou, 350122 Fujian Province China; 3 Obstetrics and Gynecology Department, Fujian Maternity and Child Hospital College of Clinical Medicine for Obstetrics & Gynecology and Pediatrics, Fujian Medical University, Fuzhou, 350001 Fujian Province China

**Keywords:** Acylcarnitine profile, GCDH, Glutaric acidemia type 1, Southeastern China, Variant

## Abstract

**Background:**

Glutaric acidemia type 1 (GA1) is a rare autosomal recessive inherited metabolic disorder caused by variants in the gene encoding the enzyme glutaryl-CoA dehydrogenase (GCDH). The estimated prevalence of GA1 and the mutational spectrum of the *GCDH* gene vary widely according to race and region. The aim of this study was to assess the acylcarnitine profiles and genetic characteristics of patients with GA1 in Fujian Province, southeastern China.

**Results:**

From January 2014 to December 2022, a total of 1,151,069 newborns (631,016 males and 520,053 females) were screened using MS/MS in six newborn screening (NBS) centers in Fujian Province and recruited for this study. Through NBS, 18 newborns (13 females and 5 males) were diagnosed with GA1. Thus, the estimated incidence of GA1 was 1 in 63,948 newborns in Fujian province. In addition, 17 patients with GA1 were recruited after clinical diagnosis. All but one patient with GA1 had a remarkable increase in glutarylcarnitine (C5DC) concentrations. The results of urinary organic acid analyses in 33 patients showed that the concentration of glutaric acid (GA) increased in all patients. The levels of C5DC and GA in patients identified *via* NBS were higher than those in patients identified *via* clinical diagnosis (*P* < 0.05). A total of 71 variants of 70 alleles were detected in patients with GA1, with 19 different pathogenic variants identified. The three most prevalent variants represented 73.23% of the total and were c.1244-2 A > C, p.(?) (63.38%), c.1261G > A, p.Ala421Thr (5.63%), and c.406G > T, p.Gly136Cys (4.22%). The most abundant genotype observed was c.[1244-2 A > C]; [1244-2 A > C] (18/35, 52.43%) and its phenotype corresponded to high excretors (HE, GA > 100 mmol/mol Cr).

**Conclusions:**

In conclusion, we investigated the biochemical and molecular features of 35 unrelated patients with GA1. C5DC concentrations in dried blood spots and urinary GA are effective indicators for a GA1 diagnosis. Our study also identified a *GCDH* variant spectrum in patients with GA1 from Fujian Province, southeastern China. Correlation analysis between genotypes and phenotypes provides preliminary and valuable information for genetic counseling and management.

## Background

Glutaric acidemia type 1 (GA1; OMIM #231670) is a rare inherited neurometabolic disorder caused by variants of the gene encoding glutaryl-CoA dehydrogenase (GCDH; EC 1.3.8.6) [1]. The *GCDH* gene is located on chromosome 19p13.2. The defective activity of GCDH hinders the degradation of L-lysine, L-hydroxylysine, and L-tryptophan, resulting in the abnormal accumulation of glutaric acid (GA) and 3-hydroxyglutaric acid (3HGA) in biological fluids and various tissues, especially in the brain [2]. Without medical management, the majority of patients with GA1 experience an acute encephalopathic crisis in the first 3–36 months following an intercurrent febrile illness or surgical intervention, resulting in bilateral striatal damage [3, 4]. The clinical presentation of striatal damage is a complex dystonic movement disorder. To avoid disease complications, patients usually receive timely medical management, including a diet low in lysine and tryptophan, supplementation with l-carnitine, and prompt complication management [5, 6]. Therefore, early diagnosis of GA1 is crucial for improved outcomes. Depending on the concentration of urinary GA, patients with GA1 are classified into two biochemical subgroups: low excretor (LE, GA < 100 mmol/mol Cr) and high excretor (HE, GA > 100 mmol/mol Cr) [7]. Several studies have assumed that HE patients have poorer outcomes than LE patients [8–11].

Increased concentrations of glutarylcarnitine (C5DC) levels in dried blood spots (DBS) and GA in urine can be reliably identified in the vast majority of patients with GA1 using tandem mass spectrometry (MS/MS) and gas chromatography/mass spectrometry (GC/MS), respectively. GA1 is a treatable disorder [1], and newborn screening (NBS) programs for GA1 have been implemented in many developed countries [12–14]. The incidence of GA1 and characteristics of the variants of *GCDH* gene have been reported in certain different populations, including the Chinese population [14–18]. However, the prevalence of GA1 ranges from 1/221,053 to 1/52,078 among the populations in different regions of China [18–22]. Limited data are available on the prevalence and mutational spectrum of *GCDH* in GA1 in China based on large-scale NBS. Although a single-center study on GA1 was conducted in Quanzhou, Fujian Province, the prevalence and genotypes of GA1 in Fujian Province, southeastern China, have not been reported.

In this study, we report the prevalence and mutational spectrum of *GCDH* in patients with GA1 based on a multicenter and large-scale NBS in Fujian Province, southeastern China. We also report the biochemical features of 18 patients with GA1 identified through NBS and 17 patients with GA1 diagnosed through clinical screening. Our work provides preliminary and valuable information for the genetic counseling and management of these patients.

## Results

### NBS for GA1

Over 12 years, 1,151,069 newborns were screened, and 265 had elevated concentrations of C5DC at initial NBS, yielding a positivity rate of 0.023%. After repeated testing, 42 newborns with positive results underwent urinary organic acid analysis and genetic testing. Additionally, 17 newborns (12 females and 5 males) were diagnosed with GA1, with a positive predictive value of 6.42% (17/265). Furthermore, one patient (no. 12) had a normal concentration of C5DC and an extremely low free carnitine (C0) level at initial NBS and was ultimately diagnosed with GA1, as shown in Table 1. As a result, the detection incidence of GA1 was 1 in 63,948 (18/1,151,069) newborns in Fujian Province.

**Table 1:** Biochemical and genetic features of 35 neonatal GA1 patients province

Patient no.	Gender	Age at diagnosis	C5DC (µmol/l)		GA (mmol/mol Cr)	Genotype		Subtype	Source
			Initial	recall		Maternal allele	Paternal allele		
1	F	43 d	2.63	4.3	1179.32	c.1244-2 A > C	c.1244-2 A > C	HE	NBS
2	M	27 d	1.5	4.19	976.87	c.1244-2 A > C	c.1244-2 A > C	HE	NBS
3	F	70 d	3.03	2.51	1094.35	c.1244-2 A > C	c.1244-2 A > C	HE	NBS
4	F	50 d	2.86	1.22	759.74	c.1244-2 A > C	c.1244-2 A > C	HE	NBS
5	F	30 d	1.61	3.09	175.7	c.1244-2 A > C	c.1244-2 A > C	HE	NBS
6	F	22 d	2.67	2.47	150.49	c.532G > A	c.108_109delAC	HE	NBS
7	F	20 d	1.45	1.5	503.79	c.533G > A	c.1244-2 A > C	HE	NBS
8	F	18 d	2.81	3.68	300.15	c.1244-2 A > C	c.1244-2 A > C	HE	NBS
9	M	25 d	2.42	N/A	409.05	c.1244-2 A > C	c.1244-2 A > C	HE	NBS
10	F	20 d	1.9	1.92	462.83	c.395G > A	c.1147 C > T	HE	NBS
11	F	21 d	3.79	N/A	N/A	c.1244-2 A > C	c.1016T > C	N/A	NBS
12	F	44 d	0.06	0.05	1.31	c.1244-2 A > C	c.1261G > A	LE	NBS
13	M	23 d	1.58	2.19	313.74	c.1244-2 A > C	c.1244-2 A > C	HE	NBS
14	M	28 d	4.26	3.34	741.81	c.1244-2 A > C	c.1244-2 A > C	HE	NBS
15	F	26 d	2.44	3.65	434.34	c.1244-2 A > C	c.1261G > A	HE	NBS
16	F	30 d	0.68	0.7	N/A	c.300G > A	c.1204 C > T	N/A	NBS
17	F	20 d	1.35	1.69	605	c.1244-2 A > C	c.1244-2 A > C	HE	NBS
18	M	25 d	1.8	2.18	741.81	c.1244-2 A > C	c.1244-2 A > C	HE	NBS
19	F	6 m	0.46	N/A	232.2	c.339delT	c.406G > T	HE	clinical diagnose
20	M	7y, 5 m	1.65	N/A	298.06	c.1147 C > T	c.1244-2 A > C	HE	clinical diagnose
21	F	3 y	0.91	N/A	135.73	c.1244-2 A > C	c.1244-2 A > C	HE	clinical diagnose
22	M	5 m	0.25	N/A	359.2	c.1244-2 A > C	c.1244-2 A > C	HE	clinical diagnose
23	F	7 m	0.26	0.36	7.45	c.533G > A	c.755G > A	LE	clinical diagnose
24	M	4y, 5 m	0.42	0.52	16.91	c.406G > T	c.1169G > A	LE	clinical diagnose
25	M	1y, 5 m	0.51	1.26	440.1	c.1244-2 A > C	c.1244-2 A > C	HE	clinical diagnose
26	M	1y, 4 m	0.97	N/A	578	c.1244-2 A > C	c.1244-2 A > C	HE	clinical diagnose
27	M	6y, 4 m	0.48	N/A	13.46	c.1244-2 A > C	c.1286 C > T	LE	clinical diagnose
28	F	6 m	1.15	N/A	242.1	c.1244-2 A > C	c.1244-2 A > C	HE	clinical diagnose
29	M	2y, 3 m	0.27	0.5	360	c.406G > T	c.881G > T	HE	clinical diagnose
30	M	1 y	0.45	N/A	14.09	c.755G > A	c.1244-2 A > C	LE	clinical diagnose
31	F	1y, 7 m	0.43	N/A	686.88	c.1244-2 A > C	c.1244-2 A > C	HE	clinical diagnose
32	M	1y, 8 m	3.2	N/A	499.16	c.1244-2 A > C	C.1261G > A	HE	clinical diagnose
33	F	2y, 2 m	4.01	N/A	985.05	c.1244-2 A > C	c.395G > T, c.1261G > A	HE	clinical diagnose
34	F	3y, 7 m	1.07	1.15	579.6	c.1244-2 A > C	c.1244-2 A > C	HE	clinical diagnose
35	F	3y, 4 m	0.76	N/A	201.5	c.1063 C > T	c.769 C > T	HE	clinical diagnose

Abbreviation: M: male, F: female, C5DC: glutarylcarnitine, GA: glutaric acid, N/A: not available, HE: high excretors, HE: low excretors, NBS: newborn screening. Reference range, C5DC: 0.03–0.2 µmol/L, GA: < 2.5 mmol/mol creatinine

### Biochemical results and clinical features

A total of 35 unrelated patients with GA1 were investigated, including 18 NBS and 17 clinical patients. The acylcarnitine concentrations showed that all but one patient had remarkably increased C5DC concentrations, with the one anomalous patient showing a normal C5DC level and an extremely low C0 level (3.18 µmol/L) during NBS. The mean C5DC in this cohort of patients was 1.60 ± 1.17 µmol/L. Additionally, 33 patients underwent urinary organic acid analyses. The results showed that the concentration of GA increased in all patients, with 28 and 5 presenting the HE and LE phenotypes, respectively (Table 1). We further compared the differences in C5DC and GA levels between patients identified *via* NBS and clinical diagnosis. The results showed that the levels of C5DC and GA in NBS patients were higher than those in clinically diagnosed patients (Table 2).

**Table 2:** Comparisons of the level of C5DC and GA and the relative frequency of c.1244-2 A > C in the GA1 patients between NBS and clinical diagnose

	C5DC (µmol/L)	GA (mmol/mol Cr)	c.1244-2 A > C (n/N, %)
NBS patients	2.16 ± 1.04	553.14 ± 342.25	26/36, 72.22
Clinical patients	1.01 ± 1.06	332.32 ± 273.55	19/35, 54.28
T or χ^2^	3.23	2.054	2.46
*P*	0.003	0.049	0.117

The clinical characteristics of all 17 patients with clinical diagnoses are summarized in Table 3. “Movement disorders” was the most common symptom, followed by seizures and mental retardation. 11 patients (64.71%) had at least one acute encephalopathic crisis, while 6 individuals (35.29%) presented with insidious onset and developed neurological disease in the absence of encephalopathic crises.

**Table 3:** Clinical features of the patients with GA1 from the clinical diagnosis

Clinical features (N = 17)	No	RF (%)
Movement disorders	9	52.94
Seizure	8	47.06
Mental retardation	7	41.17
Muscular hypotonia	6	35.29
Macrocephaly	2	11.76
Coma	2	11.76
Feeding difficulty	3	17.65
Vomiting	3	17.65
Diarrhea	2	11.76
Failure to thrive	4	23.53
Jaundice	4	23.53

Abbreviation: GA1: Glutaric acidemia type 1, RF: relative frequency

### Variant spectra of the *GCDH* gene

**Fig. 1:**
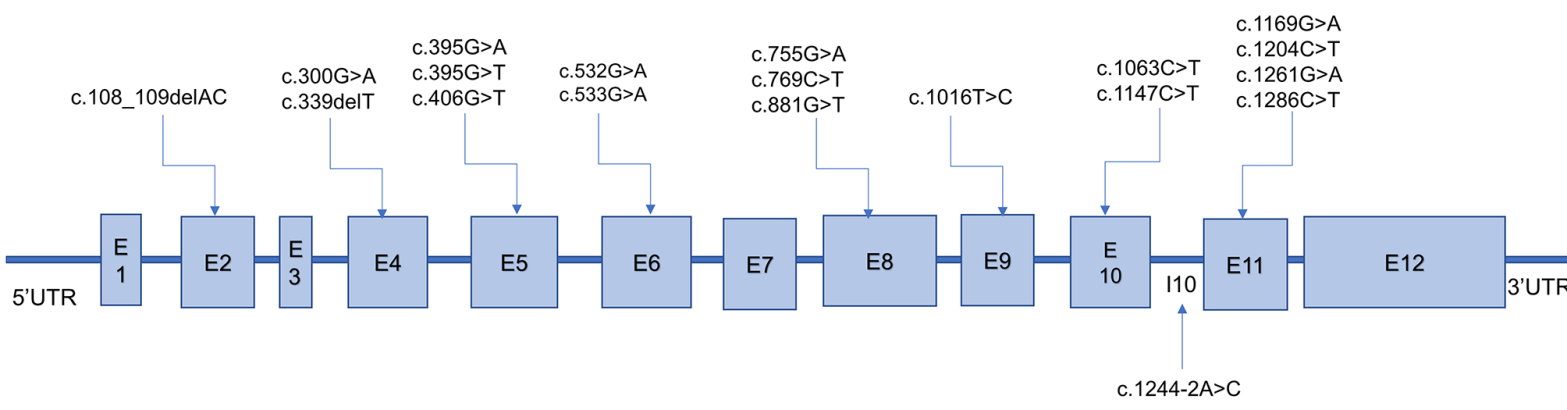
Nnineteen variants were identified in patients with GA1 from Fujian Province E: exon; I: intron. The number of *GCDH* transcription version is NM_000159.4

From the 35 patients with GA1, 71 variants of 70 alleles were detected, among which 19 different variants were confirmed, including 15 (78.94%) missense variants, 2 (10.53%) nonsense variants, 1 (5.26%) synonymous variant, and 1 (5.26%) splice-site variant (Table 4). At the amino acid sequence level, the three most prevalent variants accounted for 73.23% of the total: c.1244-2A > C, p.(?) (63.38%), c.1261G > A, p.Ala421Thr (5.63%), and c.406G > T, p.Gly136Cys (4.22%) (Table 4). Except for exons 1 and 3, the variants were relatively evenly distributed in the *GCDH* gene (Fig. 1).

**Table 4:** *GCDH* mutations in the GA1 patients from Fujian province

cDNA Aberration	Protein effect or trivial name	Gene Region	Type	Pathogenicity classification	No. Of alleles	RF (%)
c.1244-2 A > C	p.(?)	Intron 10	Splice	P	45	63.38
c.339delT	p.Thr113*	Exon 4	Nonsense	P	1	1.41
c.406G > T	p.Gly136Cys	Exon 5	Missense	LP	3	4.22
c.1261G > A	p.Ala421Thr	Exon 11	Missense	LP	4	5.63
c.395G > T	p.Arg132Ile	Exon 5	Missense	LP	1	1.41
c.1147 C > T	p.Arg383Cys	Exon 10	Missense	P	2	2.82
c.533G > A	p.Gly179Gly	Exon 6	Synonymous	LP	2	2.82
c.755G > A	p.Gly252Ala	Exon 8	Missense	P/LP	2	2.82
c.1169G > A	p.Gly390Glu	Exon 11	Missense	P	1	1.41
c.532G > A	p.Gly178Ala	Exon 6	Missense	P	1	1.41
c.395G > A	p.Ala132Gly	Exon 5	Missense	LP	1	1.41
c.108_109delAC	p.Gln37Glufs*5	Exon 2	Nonsense	P	1	1.41
c.1016T > C	p.Met339Thr	Exon 9	Missense	VUS	1	1.41
c.300G > A	p.Met100Ile	Exon 4	Missense	LP	1	1.41
c.1204 C > T	p.Ala402Thr	Exon 11	Missense	P	1	1.41
c.1063 C > T	p.Ala355Cys	Exon 10	Missense	P	1	1.41
c.1286 C > T	p.Thr429Met	Exon 11	Missense	LP	1	1.41
c.881G > T	p.Arg294Gln	Exon 8	Missense	LP	1	1.41
c.769 C > T	p.Ala257Thr	Exon 8	Missense	LP	1	1.41

Abbreviation: LP: likely pathogenic, P: pathogenic, VUS: variants of uncertain significance, RF: relative frequency, *GCDH*: glutaryl-CoA dehydrogenase, GA1: Glutaric acidemia type 1, NBS: newborn screening. The number of *GCDH* transcription version is NM_000159.4.

As c.1244-2A > C was the most prevalent variant, we further compared the differences in its frequency between the NBS and clinical diagnosis groups. The results showed that the frequency of c.1244-2A > C did not differ between the two groups (Table 2).

### Genotype of GA1 patients

Out of the 35 patients, 97.14% (34/35) carried biallelic variants and were either compound heterozygous (n = 15) or homozygous (n = 19), while the other patient (no. 33) carried a triallelic variant, with one variant originating from the mother and the other two originating from the father (Table 1). At the amino acid level, 13 distinct combinations were found in 35 patients, and the most abundant genotypes observed were c.[1244-2A > C];[1244-2A > C] (18/35, 52.43%) and c.[1244-2A > C];[1261G > A] (4/35, 11.43%) (Table 1). The phenotype of the patients with the genotype (c.[1244-2A > C];[1244-2A > C]) was HE.

## Discussion

Early detection and timely intervention for inborn metabolic disorders through NBS are crucial for preventing adverse clinical symptoms in affected individuals. With the widespread application of MS/MS, GC/MS, and NGS, patients with GA1 have been diagnosed timely [18, 23, 24]. The estimated prevalence of GA1 varies widely depending on race and region, ranging from 1:125,000 to 1:250 newborns [25–29]. Based on a national cross-sectional survey of 7 million NBS results from mainland China from 2016 to 2017, the estimated incidence of GA1 was 1:147,900 [30]. The exact prevalence of GA1 in Fujian Province, southeastern China, remains unknown. In this study, 18 patients with GA1 were identified *via* 1.15 million NBS results, and the incidence was approximately 1 in 63,948 births in Fujian Province. This incidence is much higher than that in Zhejiang Province (1:221,053) [18] and Jining City (1:171,411) in eastern China [20].

In this study, 35 unrelated patients with GA1 were identified, including 18 patients who underwent NBS and 17 who received a clinical diagnosis. All but one patient showed remarkably increased C5DC concentrations. The one anomalous patient was diagnosed with GA1 and presented normal C5CD levels and a much lower C0 level during NBS, which was suggestive of primary carnitine deficiency (PCD) or maternal PCD. However, the *SLC22A5* genetic mutation was not detected in patients *via* multi-gene-targeted NGS, although the *GCDH* genetic mutation was unexpectedly detected. Thus, it is noteworthy that, although NBS is a cost-effective strategy for identifying GA1, false negative results may still occur, especially in patients with the LE phenotype. Therefore, Newborns with persistently low C0 concentrations should be monitored for the possibility of GA1. Two patients with NBS did not undergo urine organic acid analyses because their urine could not be collected. Moreover, the increased GA concentration in 33 patients in this cohort suggests that urinary GA is an effective indicator for the diagnosis of GA1. Of note, GA1 patients with LE may have normal GA concentrations [18, 29]. Interestingly, the levels of C5DC and GA in the NBS patients were higher than those in the clinical patients, which is inconsistent with the results of a previous study [31]. We assumed that some factors led to the differences in the levels of C5DC and GA1 between the two groups. Firstly, patients with a clinical diagnosis may experience vomiting and eating difficulties that lead to lower C5DC and GA concentrations. Secondly, there may be differences in the levels of acylcarnitine among different ages, and the ages of patients with clinical diagnoses were significantly higher than those from the NBS. Additionally, the treatment that clinical patients were receiving was different from the nutrition the newborns were receiving.

More than 300 (confirmed or likely) pathogenic *GCDH* variants have been detected (http://www.hgmd.cf.ac.uk; data collected on January 1, 2023). The high-frequency region of variants and mutational spectrum of *GCDH* vary widely by race and region. Previous reports indicated that exon 11 is the most frequently mutated region in India and Brazil, accounting for approximately 50% of variants [32, 33], while another study showed that exon 8 is the most commonly mutated region in Chinese patients [31]. However, except for exons 1, 3, and 7, the variants were relatively evenly distributed in the *GCDH* gene in this study. The c.1204C > T (p.Arg402Trp) mutation is highly prevalent in European and Caucasian patients [34, 35], whereas c.914C > T (p.Ser305Leu) is a common mutation in Japanese patients [36]. Furthermore, 19 distinct variants were detected in this study, two-thirds of which were investigated only once, revealing a high degree of genetic heterogeneity among the patients with GA1 in Fujian Province. The following three variants accounted for 73.23% of the total variants among the patients with GA1: c.1244-2A > C, p.(?) (63.38%), c.1261G > A, p.Ala421Thr (5.63%), and c.406G > T, p.Gly136Cys (4.22%). Consistent with the results of previous studies [19, 31], the c.1244-2A > C variant was the predominant variant in the Chinese population. Furthermore, the c.1244-2A > C variant was more prevalent in Fujian Province than in Zhejiang Province [18].

The most prevalent genotype was c.[1244-2A > C];[1244-2A > C] (52.43%), followed by c.[1244-2A > C];[1261G > A] (11.43%), which is inconsistent with an earlier finding [18]. The phenotype of the patients with the genotype (c.[1244-2A > C];[1244-2A > C]) was HE. Compared with LE patients, HE patients show an increased risk of extrastriatal abnormalities [8] and subdural hemorrhage [9], larger macrocephaly [10], and poorer cognitive outcomes [11]. These results provide preliminary and valuable data regarding the correlation between the genotype and phenotype of GA1.

## Conclusions

In conclusion, this study investigated the biochemical and molecular features of 35 unrelated patients with GA1, including 18 patients identified *via* NBS and 17 patients identified *via* clinical diagnosis. The estimated incidence of GA1 was 1 in 63,948 newborns in Fujian Province. The concentrations of C5DC in DBS and urinary GA are effective indicators for diagnosing GA1. The levels of C5DC and GA in the NBS patients were higher than those in the clinical patients. The c.1244-2 A > C variant was the most prevalent *GCDH* variant. The most prevalent genotype was c.[1244-2A > C];[1244-2A > C] (52.43%). The phenotype of the patients with the genotype (c.[1244-2A > C];[1244-2A > C]) was HE, which can provide preliminary and valuable data for the correlation between genotypes and phenotypes.

## Methods

### Study population

From January 2014 to December 2022, 1,151,069 newborns (520,053 females and 631,016 males) were recruited for this study based on screening using MS/MS in six NBS centers in Fujian Province. In addition, 17 patients with GA1 were recruited based on clinical diagnoses.

### NBS and biochemical analysis

The workflow of NBS is based on a previously described procedure [9]. Briefly, DBS samples were collected and transported *via* a cold-chain transportation system to the corresponding NBS center. All six NBS centers used a unified experimental platform. The acylcarnitine concentrations in the DBS were quantitated using an ACQUITY TQD MS/MS (Waters) with a NeoBase^™^ MS/MS Kit (PerkinElmer, Turku, Finland). The cutoff value of C5DC was set at the 99.5th (0.05th) percentile. When the C5DC level of newborns exceeded this threshold, they underwent repeated testing. Urine samples were collected and analyzed for urine organic acids, including GA and 3HGA, using GC/MS (QP2010, Shimadzu Corp.). If the patients tested positive on the second screen, they underwent genetic analysis as a confirmatory test. Other laboratory tests were also performed to evaluate the patient’s status, including blood gas, ammonia, lactic acid, glucose, and liver and kidney function assessments.

### Genotype analysis

Genomic DNA was isolated from DBS using a QIAamp DNA Mini Kit (Tiangen Biotech, China) according to the manufacturer’s instructions. Target next-generation sequencing was performed to detect a target sequencing panel of 94 genes (including *GCDH*) related to inborn metabolic errors, as previously described [37]. Briefly, the target region sequences were enriched and purified. Thereafter, a sequencing library was constructed, and sequencing and data analysis were performed using the Illumina NextSeq 500 platform and NextSeq 500 Reporter, respectively. The detected variants were confirmed using Sanger sequencing. The variants of *GCDH* were classified according to the guidelines of the American College of Medical Genetics and Genomics (https://clinicalgenome.org/).

### Statistical analyses

Continuous variables were normally distributed and expressed as mean ± standard deviation. All categorical data are expressed as proportions. The *t*-test and chi-square test of variance were used to compare differences between the different GA1 subgroups. All statistical analyses were performed using the GraphPad Prism software (GraphPad, version 7.0). *P* value < 0.05 was considered statistically significant.

## Data Availability

All data generated or analyzed during this study are included in the article, further inquiries can be directed to the corresponding author.

## Abbreviations

GA1: Glutaric acidemia type 1
GCDH: Glutaryl-CoA dehydrogenase
NBS: Newborn screening
GA: Glutaric acid
3HGA: 3-hydroxyglutaric acid
C0: Free carnitine
C5DC: Glutarylcarnitine
DBS: Dried blood spot
NGS: Next-generation sequencing
HE: High excretion
LE: Low excretion
